# Drug 9AA reactivates p21/Waf1 and Inhibits HIV-1 progeny formation

**DOI:** 10.1186/1743-422X-5-41

**Published:** 2008-03-18

**Authors:** Weilin Wu, Kylene Kehn-Hall, Caitlin Pedati, Lynnsey Zweier, Iris Castro, Zachary Klase, Cynthia S Dowd, Larisa Dubrovsky, Michael Bukrinsky, Fatah Kashanchi

**Affiliations:** 1 The George Washington University Medical Center, Department of Biochemistry and Molecular Biology, Washington, DC 20037, USA; 2 The George Washington University, Department of ChemistryWashington, DC 20037, USA; 3 The Institute for Genomic Research, Rockville, MD 20850, USA; 4 The George Washington University, W.M. Keck Institute for Proteomics Technology and Applications, Washington, DC 20037, USA

## Abstract

It has been demonstrated that the p53 pathway plays an important role in HIV-1 infection. Previous work from our lab has established a model demonstrating how p53 could become inactivated in HIV-1 infected cells through binding to Tat. Subsequently, p53 was inactivated and lost its ability to transactivate its downstream target gene p21/waf1. P21/waf1 is a well-known cdk inhibitor (CKI) that can lead to cell cycle arrest upon DNA damage. Most recently, the p21/waf1 function was further investigated as a molecular barrier for HIV-1 infection of stem cells. Therefore, we reason that the restoration of the p53 and p21/waf1 pathways could be a possible theraputical arsenal for combating HIV-1 infection. In this current study, we show that a small chemical molecule, 9-aminoacridine (9AA) at low concentrations, could efficiently reactivate p53 pathway and thereby restoring the p21/waf1 function. Further, we show that the 9AA could significantly inhibit virus replication in activated PBMCs, likely through a mechanism of inhibiting the viral replication machinery. A mechanism study reveals that the phosphorylated p53ser15 may be dissociated from binding to HIV-1 Tat protein, thereby activating the p21/waf1 gene. Finally, we also show that the 9AA-activated p21/waf1 is recruited to HIV-1 preintegration complex, through a mechanism yet to be elucidated.

## Introduction

Amid the availability of current diverse classes of anti-HIV reagents such as reverse transcriptase (RT), protease (PR) and fusion inhibitors, the development of inhibitors targeting another important HIV enzyme IN (integrase) has stimulated great interests in that there is an absence of cellular homologues of IN in cells. A number of HIV-1 IN inhibitors have been identified and few have been clinically examined including GS-9137 [1-3] and MK-0518 [4]. Recently, increasing evidence has indicated that small chemical molecules, such as nucleoside antiretroviral reagents, may be advantageous over other antiviral reagents, since they have long intracellular half-life and low protein binding properties [5-7]. So far few detailed studies have been carried out in investigating the role of these small chemical molecules involved in the signaling pathway(s) of HIV-infected cells. However, exploring whether there are novel small chemical molecules or small peptides that can reactivate important cell signaling pathways (i.e., p53 and/or p21/waf1) that are normally inactivated in HIV-1 infected cells may help to identify important mechanisms that play key roles in HIV-1 infection and provide a new set of preventive and/or therapeutic drug-targets.

P53 pathway has been revealed to play an important role in HIV-1 infection [8,9]. It was also shown that p53 is involved in apoptosis, where cell death can be induced by HIV-1 envelope through mTOR-mediated phosphorylation of p53 on Ser15 [10,11]. Our previous work established a model where p53 was inactivated in HIV-1 infected cells through binding to Tat protein. Subsequently, p53 was inactivated and lost its ability to transactivate its downstream target gene p21/waf1 [12]. The interplay between p53 and HIV-1 Tat has also been extensively studied. An RGD-containing domain of Tat protein, Tat-(65–80), was found to be important in regulating the proliferative functions of a variety of cell lines, including a human adenocarcinoma cell line, A549. The p53 activity was greatly reduced when cells were treated with Tat-(65–80) [13]. Tat was also shown to efficiently inhibit the transcription of p53 both *in vivo *and *in vitro*. The downregulation of p53 by Tat may be important in the establishment of productive viral infection in cells and also may be involved in the development of AIDS-related malignancies [14].

The regulation of the p53 and p21/waf1 pathways by HIV-1 infection has become a point of interest. Previous studies have shown that the effects of p21/waf1 are highly cell-type specific in HIV-1 infection. In macrophages, HIV-1 infection resulted in an increased expression of p21/waf1 [15]. Repression of HIV-1 replication was observed when p21/waf1 expression was inhibited by small molecules like compound CDDO (2-Cyano-3, 12-dioxooleana-1, 9-dien-28-oic acid) [16,17]. This phenomenon was different from that of p21/waf1 in HIV-1 infection in T lymphocytes, where HIV-1 infection reduced the expression of p21/waf1 [12]. It also differs in stem cells, where silencing p21/waf1 expression by siRNA increased the viral replication [18]. Therefore, we reasoned that it is possible to inhibit the HIV-1 infection and viral replication through the restoration of p53 and p21/waf1 pathways using small chemical molecules or small peptides. More recently, the p21/waf1 function was investigated as a molecular barrier for HIV-1 infection in stem cells [18]. Hematopoietic stem cells were previously demonstrated to be highly resistant to HIV-1 infection [19-21]. In the study carried by Zhang J *et al*, the cdk inhibitor p21/waf1 was revealed to restrict HIV-1 infection in primitive hematopoietic cells. By knockdown of the endogenous p21/waf1 level using siRNAs, the stem cells became highly susceptible to HIV-1 infection. Further, it was shown that the effect of p21/waf1 is specific; silencing other p21/waf1 related proteins, p27 and p18 had no effect on HIV-1 infection. Finally, the authors demonstrated a novel mechanism in which the anti-HIV effect of p21/waf1 was the result of its interaction with HIV-1 preintegration complex (PIC). Therefore, p21/waf1 was suggested to be a possible restriction factor, similar in function to the TRIM5 and APOBEC3G genes [22-26]. Similarly, our previous work has shown that high-titer infection of HIV-1 in T lymphocytes resulted in a loss of the endogenous p21/waf1 [12], further demonstrating the importance of p21/waf1 in HIV-1 biology.

In this report, we show that a small chemical molecule 9AA can efficiently reactivate the p53 pathway in HIV-1 infected cells, and accordingly transactivates its downstream gene p21/waf1, a gene that has a potential role in inhibiting viral replication. We have also observed that the effect of 9AA on HIV-1 viral replication and virus DNA integration in HIV-1 infected cells is due to the association of 9AA-induced p21/waf1 with HIV-1 preintegration complex (PIC). The implication of these findings will be discussed below.

## Results

### Drug 9AA reactivates the p53 and p21/waf1 pathways in HIV-1 infected cells

We have previously reported that in HIV-1 infected T-cells, p53 was inactivated through binding to HIV-1 Tat protein and the expression of p21/waf1 was nearly completely inhibited as a consequence of the inactivation of p53 [12]. Small molecules, such as leptomycin B, actinomycin D, and 9AA (9-aminoacridine), were demonstrated to be able to efficiently reactivate p53 in some cancer cell lines [27-29]. Therefore, we reasoned that restoration of the p53 function may provide a new way to combat virus infection where this pathway is normally impaired or sequestered [30-32]. In this study, we specifically used 9AA and tested whether it could efficiently restore the functions of p53 and p21/waf1 in HIV-1 infected cells.

**Figure 1:**
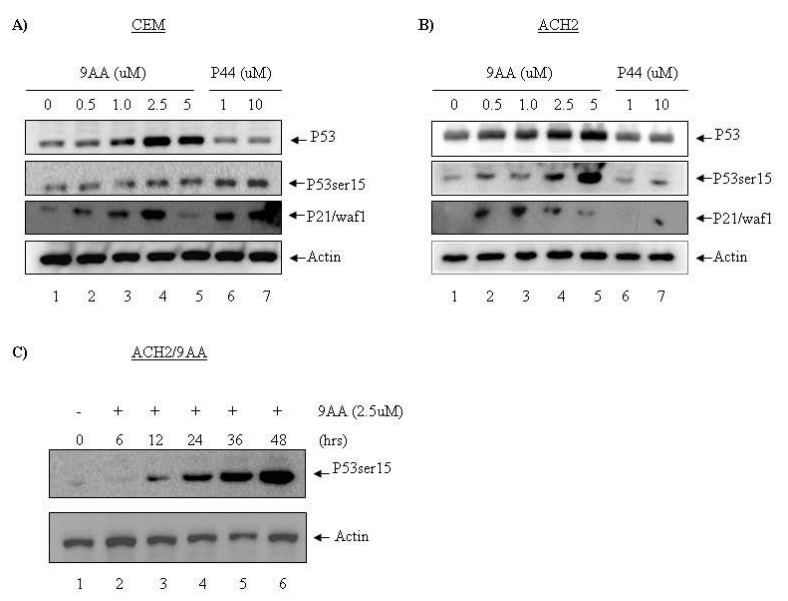
**9AA activates phosphorylation of p53 at Ser15**. CEM and ACH2 cells were treated with drug 9AA, P44 peptide or DMSO as mock control, respectively. Cells were harvested 24 hrs after treatment. Cells were then lysed and subjected to western blot for p53, p53ser15 and p21/waf1 (anti-p53, anti-p53 ser15 from Cell Signaling, anti-p21/waf1 from Santa Cruz Biotechnology). (A) CEM cells, uninfected cells. (B) ACH2 cells, HIV-1 infected cells. (C) ACH2 cells were treated with 9AA at a concentration of 2.5 uM. Cells were collected at different time points and then subjected to western blot for detection of the phosphorylation of p53ser 15. Actin was used as a loading control.

We initially started our experiments by using infected and uninfected matched latent cell lines. We used ACH2 and CEM as our model cells to study whether p53 could be reactivated with drug 9AA. We therefore treated both cell types with a titration of 9AA (0.0 to 5.0 uM). Results in Figure 1A demonstrate that uninfected CEM cells when treated with 9AA can show activation of overall p53 levels. The highest concentration of p53 levels was seen at 2.5 uM of 9AA (Figure 1A, lane 4). Also, a gradual increase of phosphorylated p53 was seen in these cells. The phosphorylation of p53 at serine 15 is a hallmark of increased DNA-binding to promoters such as p21/waf1. P21/waf1 has been shown extensively to regulate cell cycle checkpoint and apoptosis of many cell types. Further Western blots of p21/waf1 showed that it also was up-regulated by 9AA at 2.5 uM concentration (Figure 1A, lane 4). No change in actin levels were seen in any of the drug concentrations. Next, we used ACH2 cells in treating with 9AA (Figure 1B) and found that its p53 phosphorylation pattern and p21/waf1 expression was distinctly different from uninfected cells. For instance, although there was a small difference in the overall p53 levels as compared to uninfected cells, the serine 15 phosphorylation pattern was distinctly different where a gradual increase was seen up to 5.0 uM of 9AA (Figure 1B, lane 5). Also, the p21/waf1 increased levels started at 0.5 uM and peaked at 1.0 uM (Figure 1B, lanes 2 and 3) of 9AA and almost completely gone at 5.0 uM (Figure 1B, lane 5). Therefore, the pattern of p53 phosphorylation and p21/waf1 induction in infected cells is distinctly different from uninfected cells. Again, no difference in actin levels was seen in these cells. We next used a peptide (p44) that is known to activate p53 phosphorylation and induce p21/waf1 in a cdc2-dependent manner [33]. Quite surprisingly, we found that phosphorylation of p53 at serine 15 and induction of p21/waf1 occurred mainly in uninfected but not infected cells. This suggests that kinases that phosphorylate p53 in infected and uninfected cells may be quite distinct and may have altered function. It is possible that molecules such as DNA-PK and ATM may be altered by HIV-1 infection and therefore phosphorylation of its downstream molecules such as p53. Finally, we asked if p53 phosphorylation could be induced in a time-dependent manner. We added 9AA to ACH2 cells and Western blotted for phosphorylated p53 and actin. Results indicated that an increase in p53 phosphorylation is time-dependent and can be best seen at 48 hours post-treatment (Fig 1C). Collectively, these results indicate that p21/waf1 can be activated in infected cells (IC50 of ~0.25 uM) and at a higher concentration (IC50 of ~1.25 uM) in uninfected cells.

### Effect of increased p21/waf1 in infected and uninfected cells

**Figure 2:**
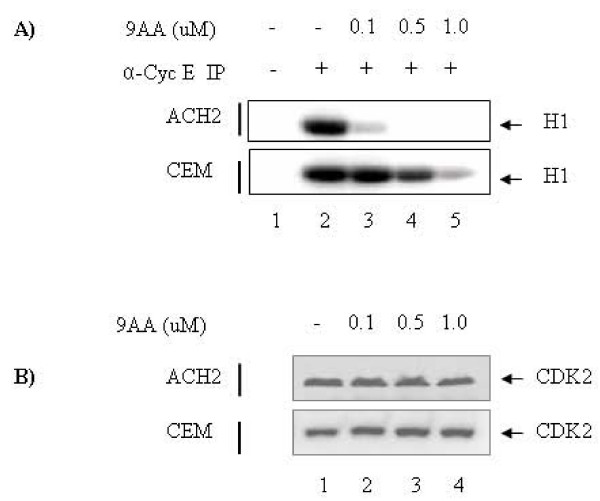
**9AA-induced inhibitory effects on cdk2/cyclin E activity in infected and uninfected cells**. (A) ACH2 and CEM cells were treated with various concentrations of 9AA (0.1, 0.5, 1.0 uM) for 24 hrs. Cells were harvested and lysed for immunoprecipitation (IP) with α-Cyc E ab followed by kinase assays. Histone H1 was used as substrate and was added to each reaction tube along with (γ-^32^P) ATP (3000 Ci/mmol). Reactions were incubated at 37°C for 30 minutes and stopped by the addition Laemmli buffer. The samples were then separated on a 4–20% Tris-Glycine gel. The gel was dried and exposed to a PhosphorImager cassette and analyzed utilizing Molecular Dynamic's ImageQuant Software. (B) The lysates from (A) were subjected to western blot to evaluate the levels of cdk2 in samples treated with 9AA at different concentrations (0, 0.1, 0.5, 1.0 uM).

We have previously shown that drugs or peptides that increase levels of p21/waf1 result in down-regulation of cdk2/cyclin E kinase activity [34]. To determine the *in vivo *binding and the kinase activity of cdk2/cyclin E to p21/waf1, we treated both CEM and ACH2 cells with various concentrations of 9AA (0.1, 0.5, 1.0 uM). We treated these cells for 24 hours and subsequently lysed and used the extract for immunoprecipitation (IP) with anti-cyclin E antibody. The rationale here is that if p21/waf1 is produced after drug treatment, it could subsequently complex with cdk2/cyclin E decreasing its kinase activity. Cyclin E IPs were used for *in vitro *kinase activity assay using histone H1 as substrate. Results in Figure 2A indicate that kinase activity in both cell types was nearly identical as seen in lane 2. Cells treated with increasing concentration of 9AA showed that cdk2/cyclin E activity was dramatically reduced in HIV-1 infected cells at 0.1 uM (Figure 2A, lane 3). However, a dramatic decrease of kinase activity was seen in uninfected cells at 1.0 uM (Figure 2A, lane 5). Beads alone control did not bring down cdk2/cyclin E or any other kinase to phosphorylate the histone H1 (Figure 2A, lane 1, negative control). Importantly, cdk2 levels were not changed upon the treatment at different concentrations of 9AA both in ACH2 and CEM (Fig 2B). Collectively these data indicate that an increase in p21/waf1 in infected cells at low concentrations was capable of sequestering cdk2/cyclin E activity.

### Effect of 9AA in PBMC infected cells

**Figure 3:**
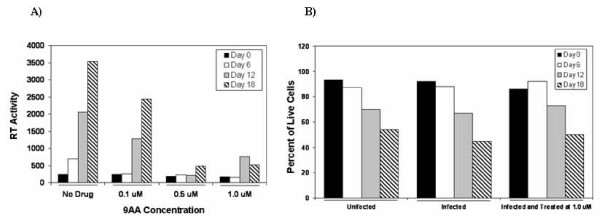
**9AA inhibits HIV-1 viral replication in PBMCs**. Phytohemagglutinin-activated PBMCs were kept in culture for 2 days prior to infection. Isolation and treatment of PBMCs were performed by following the guidelines of the Centers for Disease Control. Approximately 5 × 10^6 ^PBMCs were infected with pNL4 (MOI: 1.0). 9AA treatment (0, 0.1, 0.5 and 1.0 uM) was performed immediately after the addition of fresh medium. (A) Samples were collected every 6th day and stored at -20°C for RT assays. (B) Cells were also counted (~100/date) for viability using trypan blue staining.

To detect whether 9AA could indeed function as an inhibitor of HIV-1, we utilized a PBMC infection *in vitro*. PHA and IL2 stimulated PBMCs were infected with NL4-3 virus at an MOI of 1.0. Cells were subsequently treated with 9AA at various concentrations including 0.1, 0.5, and 1.0 uM. Cells were maintained up to 18 days in complete media in the presence of IL2. Subsequently supernatants that were collected at days 0, 6, 12, and 18 were assayed for the presence of RT. Results are shown in Figure 3. Panel A indicates that viral infection can effectively be blocked at 0.5 uM although 0.1 uM had ~30–40% inhibitory effect. Therefore, the IC50 is at ~0.25 uM for these PBMC infected cells. More importantly, viability assays of PBMC infected cells showed no difference as compared to infected alone or uninfected cells (Figure 3B). Results with 1.0 uM treatment of PBMCs showed a similar pattern of overall cell death when compared to uninfected cells. Collectively these data indicate that low concentration of 9AA that is not toxic to primary cells can effectively inhibit HIV-1 replication *in vitro*.

### Effect of phosphorylation of serine 15 p53 on Tat binding

**Figure 4:**
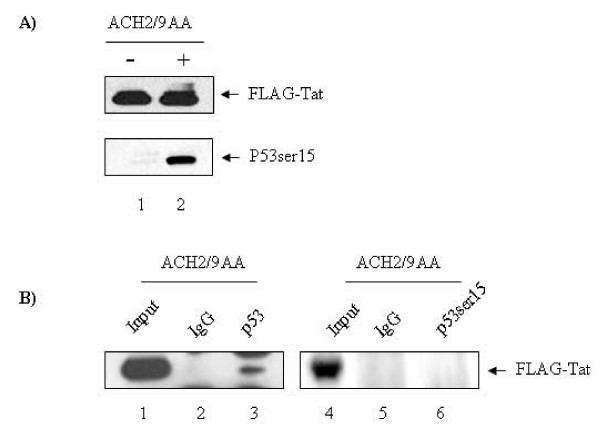
**Phosphorylated p53ser15 doesn't interact with HIV-1 Tat protein**. (A) ACH2 cells were transfected with FLAG-Tat and treated with 9AA or DMSO as a mock control. Expression of FLAG-Tat was detected by anti-FLAG (Sigma). Reactivation of p53ser15 was evaluated by anti-p53ser15 (Cell Signaling). (B) The cell lysates were then used for immunoprecipitation with anti-p53 or anti-p53ser15 (Cell Signaling). The immunopreciptated complexes were separated on 4–20% SDS-PAGE gels and then submitted to western blot to detect the presence of FLAG-Tat.

We have previously shown that unmodified Tat binds directly to p53 [12]. This is in agreement with a number of other publications that showed similar Tat p53 binding [12,13,35-39]. We now asked whether drug treatment which results in phosphorylation of p53 could still show binding to Tat. Therefore we transfected ACH2 cells with a Flag-Tat plasmid and looked for the presence of Flag-Tat and phosphorylated p53 in treated and untreated cells. Results in Figure 4A show that Flag-Tat and phospho p53 could be detected before drug treatment. Importantly, 9AA treatment of these cells did not alter the expression level of Flag-Tat but greatly increased serine 15 p53 levels. We next immunoprecipitated serine 15 p53 and asked if Tat was present in that complex after drug treatment. Results in panel B show that serine 15 phosphorylated p53 has been dissociated away from Tat and therefore may now be free to bind to endogenous promoters such as p21/waf1. In contrast, Tat was found to be associated with the p53 when the same lysates were incubated with anti-p53, which is in agreement with our previous work that p53 is inactivated though binding to HIV-1 Tat protein [12]. Collectively these results indicate that phosphorylation of p53 affects its release from Tat and its DNA-binding activity and ultimately induce gene expression on promoters such as p21/waf1.

### Drug 9AA induces p21/waf1 and its recruitment into pre-integration (PIC) complex

**Figure 5:**
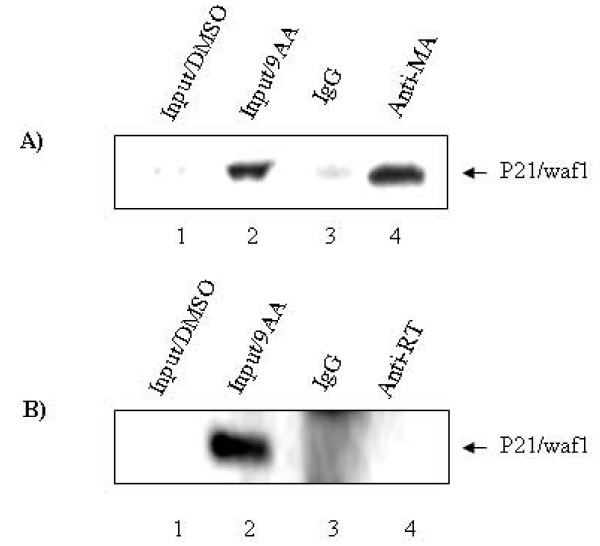
**P21/waf1 is recruited to HIV-1 preintegration complex (PIC)**. ACH2 cells were treated with 9AA or DMSO as mock control. Cells were then harvested, lysed and submitted to immunoprecipitation with anti-MA or anti-RT (ABI Inc., Columbia, MD). The immunopreciptated complexes were separated on 4–20% SDS-PAGE gels and then submitted to western blot to detect the presence p21/waf1. P21/waf1 is found to be present in the anti-MA immunoprecipitation complex.

A recent publication by Zhang J. *et al *[18] has shown that p21/waf1 is a significant barrier of HIV-1 replication in stem cells. These investigators showed that the addition of siRNA against p21/waf1 (which was normally present at high levels) in stem cells allowed active replication of HIV-1 in these cells. They also suggested that the p21/waf1 could be complexed with the HIV-1 PIC complex therefore inhibiting the integration of HIV-1 DNA into the chromosome. Inspired by their work, we asked if p21/waf1 levels induced by 9AA could also bind to pre-integration complex (matrix protein) in our latent cells. Therefore, ACH2 cells were treated with 9AA and subsequently immunoprecipitated with anti-matrix protein. Results in Figure 5A show that p21/waf1 was indeed associated with matrix protein in these cells after 9AA treatment. Anti-RT (Reverse Transcriptase) immunoprecipitation was included in this experiment. We found that p21/waf1 was not present in the anti-RT immunoprecipitated complex, which demonstrates that p21/waf1 is specifically associated with HIV-1 MA (Figure 5B). Collectively these data indicate that p21/waf1 may indeed bind to pre-integration complex provided that cells are first treated with 9AA prior to integration, expanding the role of p21/waf1 molecule not only in inhibiting integration but also transcription as previously shown [12].

### 9AA-treatment involved in post-reverse transcriptional processes of HIV-1 infection

To further explore the mechanism of the antiviral action of 9AA, we designed experiments to examine whether 9AA affects the reverse transcriptional process and/or post-reverse transcriptional process. To this end, CEM cells were infected with HIV-1 for 6 hrs. The infected cells were then washed with PBS and cultured with complete medium containing 9AA (1.0 uM) or DMSO as mock control. From our analysis of the viral copies by QPCR, we found that 9AA did not affect the entry step of HIV-1, which was evident by the fact that viral copies were observed in the HIV-1 infected/+9AA-treated samples at the 24 hrs time point. However, samples that were collected after 48 and 72 hrs, demonstrated a significant decrease of viral copy numbers in the infected/+9AA-treated samples as compared to HIV-1/+DMSO-treated sample (Table 1). Collectively, these results indicated that the drug 9AA does not affect the entry step of virus; instead, 9AA may also affect the steps after reverse transcription, mostly probably the integration step.

**Table 1:** Viral mRNA levels present in CEMs under different treatments

Treatment	mRNA level (24 hrs)	mRNA level (48 hrs)	mRNA level (72 hrs)
HIV-1+DMSO	35.8	63.4	340.3
HIV-1+ 9AA	21.7	16.2	21.7

CEM cells were infected with HIV-1 for 6 hrs before treatments with DMSO/or drug 9AA. The CEMs with different treatments were harvested at three time points: 24, 48 and 72 hrs, respectively. Viral mRNA levels were analyzed by qPCR.

## Discussion

P53 was previously shown to be inactivated by HIV-1 infection in T-cells, and consequently downregulates the expression of its target gene p21/waf1 [12]. In this study, we demonstrated that the function of p53 and p21/waf1 pathways could be restored by using a small chemical molecule 9-aminoacridine (9AA) (Figure 1). Very interestingly, 9AA was shown to differentially trigger the activation of p53 in HIV-1 infected and uninfected cells (Figure 1). P53 is present at low levels under unperturbed conditions, but it becomes rapidly activated and stabilized upon induction by a number of stimuli, including the use of reagents that cause DNA damage [40-45]. Phosphorylation plays a critical role in the activation and stabilization of p53. Of particular interest is the phosphorylation of ser15, which is generally considered to be activated in response to different stress signals [46-50]. The p53 pathway has been demonstrated to play a key role in HIV-1 infection [8,9]. Previous work in our lab has established a model demonstrating how p53 could become inactivated in HIV-1 infected cells through binding to Tat. P53 was inactivated and lost its ability to transactivate its downstream target gene p21/waf1 [12]. In our current study, we show that the 9AA-triggered phosphorylated p53ser15 does not interact with HIV-1 Tat protein. One possible explanation may be that the p53ser15 is located in the core pocket domain which is required for the p53-Tat interaction, while the phosphorylation of ser15 greatly reduces the binding affinity to Tat protein.

In the current study we propose that it is feasible to reduce the HIV-1 infection and viral replication through the restoration of p53 and p21/waf1 pathways by using small chemical molecules or small peptides. When HIV-1 PBMCs were treated with 9AA at a concentration-dependent manner (0, 0.01, 0.5, 1.0 uM), the viral replication was significantly inhibited at 0.5 uM (Figure 3A), while the cell growth was not greatly affected (Figure 3B). Further, we performed an *in vitro *kinase assay with another HIV-1 positive cell line ACH2 treated with 9AA. We immunoprecipited cdk2/cycle E complex from the drug treated and untreated samples and the results show that 9AA induced an inhibition of the kinase activity of cdk2/cycle E complex (Figure 2), indicating that the HIV-1 infected cell line(s) may be more sensitive to the drug treatment, as compared to the HIV-1 negative cell line(s).

P21/waf1 has been shown to have pleiotropic functions that are cell-type specific [51-53]. Most recently, the p21/waf1 function was identified as a molecular barrier for HIV infection of stem cells [18]. Zhang J *et al *have demonstrated a novel mechanism in which the anti-HIV effect of p21/waf1 was the result of its interaction with HIV-1 preintegration complex (PIC). Therefore, p21/waf1 was suggested to be a possible restriction factor, similar in function to the TRIM5 and APOBEC3G genes [22-26]. Consistent with this notion we have shown that the inactivated signaling pathways p53 and p21/waf1 by HIV-1 infection can be restored by a small molecule 9AA. Further, the 9AA-induced p21/waf1 was found to be recruited to HIV-1 PIC. Interestingly, we found the small molecule 9AA also inhibits the viral DNA integration step, which indicates that the drug 9AA is involved in the late stage of HIV-1 infection, rather than the early stages of infection.

## Conclusion

In our current study, we have shown for the first time a functional restoration of the important signaling pathway(s) inactivated by HIV-1 infection using small chemical molecules. Further, our study also revealed a molecular mechanism by which the 9AA-induced inhibition of HIV-1 virus replication. It would be of great interest to carry out a future screening of a large number of chemically synthesized 9AA analogs, through which we may be able to identify more effective components in activating the p53 and p21/waf1 pathways, and in inhibiting virus replication at low concentrations. Therefore our results may provide a novel therapeutical arsenal for combating HIV-1 infection.

## Materials and methods

### Plasmids, drugs and antibodies

Flag-Tat plasmid was obtained from Dr. Hiscott J. (McGill, Montreal). Drug 9-aminoacridine (9-AA) and DMSO was purchased from Sigma (Cata.Nr. 06650). P44 peptide was synthesized from GenScript Corporation (Piscataway, USA). Anti-p53 and anti-p53ser15 were purchased from Cell Signaling; anti-FLAG was purchased from Sigma; anti-p21/waf1 and anti-Actin were purchased from Santa Cruz Inc.

### Cell culture, transfections and drug treatments

ACH2, CEM and PBMC cells were maintained in RPMI-1640 medium supplemented with 10% FBS, L-glutamine (2 mM), and penicillin (100 U/ml)/streptomycin (100 μg/ml) (Quality Biological). For transfection of FLAG-Tat plasmids into ACH2 cells, five millions ACH2 cells were transfected with 5 μg of plasmid by nucleofection according to the manufacturer's protocol (Amaxa, Cologne, Germany). The cells were then incubated for 6 hrs before treatment with 9AA or DMSO as mock control. Twenty four hrs after drug or DMSO treatment, the cells were harvested for evaluation of the Tat expression and IP experiments with specific antibodies.

### Cell viability assays

After the indicated time of drug treatment, the cells were harvested and stained by Trypan blue. The viable cell number was normalized with control group and the results were expressed as relative cell viability. To evaluate the effects of 9AA on long-term growth, we collected PHA+IL-2 activated PBMC cells at different time-points, 0, 6, 12 and 18 days and stained for viability.

### Co-immunprecipitations

Cells were harvested at 4°C and cell pellets were washed with Dulbecco's phosphate-buffered saline (PBS). Cell lysates were prepared as previously described [18]. Five micrograms of Anti-MA (ABI Inc., Columbia, MD) were incubated with 2 mg of whole cell lysates overnight at 4°C with rotation. The overnight-incubated mixture was then cleared by centrifugation and Protein A/G beads (30% slurry) were added for 2 h at 4°C. The immunoprecipitated complex was washed with buffer K (150 mM KCl, 20 mM HEPES, pH 7.4, 5 mM MgCl_2_), then resuspended in SDS-PAGE loading laemmli buffer. Samples were separated on a 4–20% SDS/PAGE gel and subjected to western blot.

### Western blotting analysis

For SDS-PAGE and western blotting of p53, p21/waf1 and Tat, total cellular proteins were prepared with ice-cold lysis buffer (50 mM Tris, 5 mM EDTA, 0.1% Triton X-100, 150 mM NaCl and mixed cocktail protease inhibitors). Cell debris was removed by centrifugation, the supernatants were collected and the protein concentrations were determined by protein quantification kit (Bio-Rad, CA). Protein samples were separated on 4–20% Tris-glycine gels (Invitrgen), and transferred on PVDF membranes. Anti-p53, anti-p53ser15 (Cell Signaling) and anti-p21/waf1 were used for immunodetection (Santa Cruz).

#### RT Assays

Reverse transcriptase assay was performed according to a standard procedure. In brief, 10 ul of cell free supernatant was incubated in RT buffer (0.2 M Tris-HCL, 0.2 M DTT, 0.2 M MgCl_2_, 0.2 M KCL) in the presence of 0.1% Triton X-100, PolyA template, PolyD(T) primer and ^3^HTTP overnight at 37°C. After incubation 5 ul of the reaction mix was spotted on a DEAE filter and allowed to dry. Excess ^3^HTTP was removed by four washes with 5% Na_2_HPO_4 _followed by rinsing with water. Incorporation of ^3^HTTP was measured using a scintillation counter. RT activity is measured as CPM according to the scintillation readout.

### Quantitative real-time PCR

CEM (12D7) cells were infected with HIV-1 LAI and followed by 9AA or DMSO treatments. Cells were harvested at different time-points, 24, 48, 72 hrs. Total DNA was isolated by DNAzol^® ^Genomic DNA Isolation Reagent according to their instruction, and analyzed by Real-Time PCR using the TaqMan method with primers and probes specific for late reverse transcripts. Products were amplified from 10 μl of DNA in 50 μl reactions containing 1 × TaqMan Universal PCR Master Mix, 300 nM primers and 100 nM probe with primers: FOR-LATE (5'-TGTGTGCCCGTCTGTTGTGT-3'), REV-LATE (5'-GAGTCCTGCGTCGAGAGAGC-3') and the probe (5'-/56-FAM/CAGTGGCGCCCGAACAGGGA/36-TAMTph/-3) (Integrated DNA Technologies, Inc).

#### Kinase Assays

ACH2 cells treated with 9AA or DMSO were harvested in vitro kinase assay. Kinase assay was performed after immunoprecipitatting with anti-cycle E ab from 2 mg of ACH2 or CEM cells treated with 9AA or DMSO as mock control. The kinase assay was performed according to method described previously [54]. Phosphorylated substrate Histone H1 was resolved on 4–20% Tris-glycine gel, dried and then subjected to autoradiography (Packard Instruments, Wellesley, MA).

## Competing interests

The author(s) declare that they have no competing interests.

## Authors' contributions

WW and FK participated in the experimental design and performed the experiments for most of the data presented in this manuscript. KK, CP, LZ, LC, ZK, CSD, LD, and MB partially participated in the experimental design or manuscript revision. FK conceived the project, participated in the experimental design and manuscript revision, and is corresponding author. Authors read and approved the manuscript.
